# Evaluation of the protective efficacy of a recombinant adenovirus-vectored SARS-CoV-2 vaccine candidate for veterinary use

**DOI:** 10.3389/fcimb.2025.1714427

**Published:** 2025-12-17

**Authors:** Chong Wang, Zhiyuan Wen, Gongxun Zhong, Lei Shuai, Chen Wang, Qilong Liu, Chenyu Ren, Jinying Ge, Xijun Wang, Jinliang Wang, Renqiang Liu, Xianfeng Zhang, Yuntao Guan, Xijun He, Zhigao Bu

**Affiliations:** 1 State Key Laboratory for Animal Disease Control and Prevention, Harbin Veterinary Research Institute, Chinese Academy of Agricultural Sciences, Harbin, China; 2 National High Containment Laboratory for Animal Diseases Control and Prevention, Harbin, China; 3 Jiangsu Co-innovation Center for Prevention and Control of Important Animal Infectious Diseases and Zoonoses, Yangzhou, China

**Keywords:** veterinary vaccine, SARS-CoV-2, adenovirus vector, protective efficacy, animal models

## Abstract

Since 2019, Severe acute respiratory syndrome coronavirus 2 (SARS-CoV-2) has posed a global health threat. Its high transmissibility and cross-species infectivity have disrupted public health systems and worldwide economies, with companion and agricultural animals, like cats and minks, showing high susceptibility. ​This study evaluated rAd5-S6P, a recombinant vaccine using an adenovirus type 5 vector expressing a modified SARS-CoV-2 spike protein, in murine, feline, and mink models. Results demonstrated that rAd5-S6P elicited a robust humoral immune response when administered via intramuscular, intranasal, and oral routes, which conferred complete protection in mice. In feline and mink models, immunization with rAd-S6P resulted in significantly reduced viral shedding after high-dose SARS-CoV-2 challenge, and no infectious virus was detected in any of the examined organs of minks. Collectively, rAd5-S6P exhibited protective efficacy across species, supporting its translational potential as a veterinary vaccine. These findings provide critical evidence for animal vaccination strategies to control SARS-CoV-2 circulation and reduce zoonotic transmission risks.

## 1 Introduction

Since its emergence in December 2019, coronavirus disease 2019 (COVID-19) has rapidly become a global pandemic, imposing substantial social and economic burdens and profoundly affecting both human and animal health. Severe acute respiratory syndrome coronavirus 2 (SARS-CoV-2), the causative agent, is highly contagious and can induce severe respiratory distress syndrome and pulmonary lesions in humans.

The natural reservoir of SARS-CoV-2 remains elusive. However, accumulating evidence indicates a broad mammalian host range besides bats that includes pangolins, domestic cats, dogs, ferrets, minks, raccoon dogs, cattle, tigers, lions, white-tailed deer, and non-human primates (Younes et al., 2020; Palmer et al., 2021; Hale et al., 2022). Deer mice are susceptible to infection, with viral replication occurring in both the upper and lower respiratory tracts (Griffin et al., 2021). Domestic cats, minks, and ferrets exhibit heightened susceptibility, frequently developing significant pulmonary pathology and shedding high viral loads (Shi et al., 2020; Shuai et al., 2021). Under experimental conditions, infected cats can transmit the virus via aerosol droplets or direct contact (Shi et al., 2020). Furthermore, studies have demonstrated that cats owned by COVID-19 patients exhibit a higher seroconversion rate (Hamer et al., 2021), indicating their potential role as intermediate hosts of SARS-CoV-2. Collectively, these characteristics facilitate intra-species transmission and pose a substantial risk of spillover to humans (Oreshkova et al., 2020; Shi et al., 2020). Minks are highly susceptible to SARS-CoV-2 infection, with the virus demonstrating efficient replication in both the upper and lower respiratory tracts. SARS-CoV-2 transmits efficiently among minks via respiratory droplets, and evidence of zoonotic mink-to-human transmission—coupled with human-to-human spread of virus strains derived from minks—prompted governments in the Netherlands and Denmark to order the culling of millions of minks (Oude Munnink et al., 2021; Dall Schmidt and Mitze, 2022). As companion or agricultural animals with frequent human contact, they can act as intermediate hosts, increasing exposure risk and providing an environment for viral mutation. Anthroponotic transmission has been documented in minks (Oude Munnink et al., 2021), cats (Pagani et al., 2021), dogs (Decaro et al., 2021), tigers, and lions (McAloose et al., 2020), with reverse zoonotic events also reported (Sila et al., 2022; Yen et al., 2022). Given these dynamics, developing effective vaccines and antivirals for susceptible animal species is paramount importance.

The spike (S) protein is central to antiviral immunity, as neutralizing antibodies against it can inhibit viral replication, making it a primary vaccine target. A growing body of evidence indicates that the ancestral isolate of SARS-CoV-2 can infect various animal species, replicate in both the upper and lower respiratory tracts, and shed infectious virus in nasal washes, oropharyngeal swabs, and rectal swabs (Shi et al., 2020; Griffin et al., 2021; Shuai et al., 2021). Therefore, we used the ancestral isolate as a representative strain and its S protein as the immunogen.

Recombinant adenovirus type 5 (Ad5)-vectored SARS-CoV-2 S protein vaccines have shown robust immunogenicity in human trials, inducing high titers of neutralizing antibodies that protect against severe disease (Wu et al., 2020; Zhu et al., 2020b; Zhu et al., 2020a; Zhu et al., 2022). Furthermore, mutating six specific amino acid residues (817F, 892A, 899A, 942A, 986K, 987V) of the S protein to proline (P) maintains antigenicity and conformation while enhancing expression and stability (Hsieh et al., 2020). Building on this, we incorporated these six proline mutations into the S protein and cloned the modified gene into a replication-defective human adenovirus serotype 5 vector, generating a novel vaccine candidate. We then conducted immunogenicity and challenge experiments in mice, cats, and minks to evaluate its potential as a veterinary vaccine.

## 2 Materials and methods

### 2.1 Facility, ethics, and biosafety statement

All experiments involving infectious SARS-CoV-2 were conducted in Biosafety Level 4 (BSL-4) and Animal Biosafety Level 4 (ABSL-4) facilities at the Harbin Veterinary Research Institute (HVRI), Chinese Academy of Agricultural Sciences (CAAS). These facilities are approved for such work by the Ministry of Agriculture and Rural Affairs of China. Animal studies were performed in strict accordance with the *Guide for the Care and Use of Laboratory Animals* issued by the Ministry of Science and Technology of the People’s Republic of China. All protocols were reviewed and approved by the Animal Ethics Committee of HVRI, CAAS (Ethical Approval No. 220418-02).

### 2.2 Cells and viruses

Vero E6 cells (ATCC CRL-1586) were maintained in Dulbecco’s Modified Eagle’s Medium (DMEM) supplemented with 10% fetal bovine serum (FBS) and antibiotics, and incubated at 37 °C in a 5% CO_2_ atmosphere. AD-293 cells, an adenovirus packaging cell line complementing the E1 gene deleted from human adenovirus serotype 5, were cultured in DMEM containing 10% FBS under identical incubator conditions. SARS-CoV-2/HRB25/human/2020/CHN (HRB25, GISAID accession EPI_ISL_467430) was isolated from a patient sample using Vero E6 cells (Wang et al., 2020). Viral stocks prepared in Vero E6 cells using DMEM supplemented with 5% FBS. The mouse-adapted strain SARS-CoV-2/HRB26/human/2020/CHN (HRB26M, GISAID accession EPI_ISL_459910) was derived from a patient isolate and serially passaged in 4–6-week-old mice for 14 generations (Wang et al., 2020). Infectious virus titers were determined by plaque-forming unit (PFU) assay in Vero E6 cells.

### 2.3 Quantitative real-time PCR

Viral genomic RNA from SARS-CoV-2 was extracted using the QIAamp Viral RNA Minikit (Qiagen, Hilden, Germany). Reverse transcription for qPCR was performed using HiScript II Q RT SuperMix (Vazyme, Nanjing, China). Quantitative PCR was conducted to quantify viral *N* gene RNA copies using the Applied Biosystems QuantStudio 5 Real-Time PCR System (Thermo Fisher Scientific, Waltham, MA, USA) with Premix Ex Taq (Probe qPCR) (Takara, Dalian, China). Primers specific to the *N* gene (forward: 5’-GGGGAACTTCTCCTGCTAGAAT-3’; reverse: 5’-CAGACATTTTGCTCTCAAGCTG-3’) and a corresponding probe (5’-FAM-TTGCTGCTGCTTGACAGATT-TAMRA-3’) were used as described by the National Institute for Viral Disease Control and Prevention, China (http://nmdc.cn/nCoV). The quantity of the target SARS-CoV-2 *N* gene vRNA was normalized to a standard curve generated using a plasmid (pBluescriptIISK-N) containing the full-length cDNA of the SARS-CoV-2 *N* gene.

### 2.4 Plaque reduction neutralization test

The plaque reduction neutralization test (PRNT) was performed as previously described (Daniels et al., 1961). SARS-CoV-2 HRB25 strain (100 PFU) was incubated with two-fold serially diluted sera for 1 hour at 37°C. Subsequently, plaque assays were performed in Vero E6 cells using these neutralization mixtures. Neutralizing antibody titers were defined as the highest dilution of serum that achieved a 50% reduction in plaque count relative to the control serum.

### 2.5 Vaccine preparation

A recombinant adenovirus expressing the SARS-CoV-2 HRB25 S protein was generated using the AdEasy XL Adenoviral Vector System (Stratagene, USA). The *S* gene of the SARS-CoV-2 strain HRB25 was synthesized and subjected to mammalian codon optimization to enhance expression efficiency. Six specific amino acid residues in the S protein (817F, 892A, 899A, 942A, 986K, and 987V) were mutated to proline, and the native signal peptide was replaced with the tissue plasminogen activator (tPA) signal peptide. The modified *tPA-S6P* gene was cloned into the pShuttle-CMV shuttle vector and integrated into the adenovirus genome via homologous recombination with the pAdEasy-1 vector in *E. coli*. After linearization, the recombinant adenoviral plasmid was transfected into AD-293 cells to rescue the recombinant adenovirus (rAd-S6P), which was subsequently passaged, titrated, and aliquoted for storage.

The 50% tissue culture infectious dose (TCID_50_) of rAd-S6P was determined by infecting AD-293 cells with 10-fold serial dilutions of the virus. Titers were calculated using the Reed-Muench method (Reed and Muench, 1938).

### 2.6 Indirect immunofluorescence assay

AD-293 cells were infected with the recombinant virus rAd-S6P at a multiplicity of infection (MOI) of 0.01, with uninfected cells included as mock controls. After 48 hours of incubation at 37 °C, fluorescent staining was performed using a rabbit anti-SARS-CoV-2 S protein polyclonal antibody (Sino Biological, Beijing, China) as the primary antibody, and a fluorescein isothiocyanate (FITC)-conjugated goat anti-rabbit IgG as the secondary antibody. Images were captured using a laser scanning confocal microscope.

### 2.7 Western blotting

To verify the expression of the S protein in the recombinant virus rAd-S6P, cell lysates were prepared from AD-293 cells infected with either rAd-S6P or wild-type rAd. These lysates were subjected to SDS-PAGE separation followed by Western blotting analysis. A rabbit anti-SARS-CoV-2 S protein polyclonal antibody (Sino Biological, Beijing, China) was used as the primary antibody, and a horseradish peroxidase (HRP)-conjugated goat anti-rabbit IgG served as the secondary antibody. Signals were detected using a chemiluminescence assay.

### 2.8 Mouse experiment

Six groups of 4–6-week-old Balb/c mice were used in the study. Each group (n = 10) received three doses via three different routes: intramuscular (IM) injection at 1×10^8.5^, 1×10^7.5^, or 1×10^6.5^ TCID_50_ per 100 μL per mouse; intranasal (IN) drip at 5×10^6.5^ TCID_50_ per 50 μL per mouse; or oral administration at 1×10^7.5^ TCID_50_ per 100 μL per mouse. All immunized groups received booster vaccinations at 3-week intervals, whereas 10 control mice were administered IM injections of PBS (100 μL/mouse).

Blood samples were collected from the infraorbital venous plexus at 3 weeks post-primary immunization and 2 weeks post-booster immunization. At each time point, five mice were randomly selected from each group. Sera from mice within the same group were pooled for analysis of S protein–specific IgG and neutralizing antibodies.

Two weeks after the second immunization, mice were challenged intranasally with 10^3.6^ PFU of the mouse-adapted SARS-CoV-2 strain HRB26M, as previously described (Wang et al., 2020). SARS-CoV-2 neutralizing antibodies were measured prior to the second immunization and prior to challenge. On days 3 and 5 post-challenge, three mice from each group were anesthetized via inhalation of isoflurane (RWD, China) and subsequently euthanized by cervical dislocation. Nasal turbinate and lung tissues were then harvested to quantify viral RNA and determine the titers of infectious virus. The remaining immunized mice in each group that were not subjected to viral challenge were euthanized.

### 2.9 Cat immunization and challenge study

For the cat immunization and challenge study, twelve juvenile cats (5–6 months old) were used. Six cats were intramuscularly administered 1 mL of PBS serve as the sham control group, while the remaining six cats received two IM immunizations with rAd-S6P (1×10^9.5^ TCID_50_ per dose) at a 3-week interval. Four weeks after the second immunization, both the immunized group and the control group (with three cats per group) were challenged via IN inoculation with HRB25 at a dose of 5×10^6^ PFU/mL. Nasal wash samples were collected on days 2, 4, 6, 8, and 10 post-challenge for viral RNA and infectious virus detection to monitor the dynamics of viral shedding. Additionally, SARS-CoV-2 neutralizing antibodies levels in immunized cats were quantified prior to the second immunization and immediately before the challenge.

### 2.10 Mink immunization and challenge studies

Nine 13-month-old minks were intramuscularly inoculated with rAd-S6P at a dose of 1×10^9.5^ TCID_50_ per animal in a total volume of 1 mL. Additionally, nine minks in the control group received 1 mL of PBS via IM injection. A booster immunization was administered 3 weeks after the primary vaccination. All minks were observed daily for clinical signs of disease. Venous blood samples were collected at 3 weeks post-primary immunization and 2 weeks post-booster immunization. Sera were isolated, and neutralizing antibodies against SARS-CoV-2 were quantified using a PRNT.

For assessing the protective efficacy of rAd-S6P, three immunized minks and three PBS-inoculated control minks were intranasally challenged with the SARS-CoV-2 HRB25 strain two weeks after booster immunization, at a dose of 1×10^7^ PFU/1 mL per animal. Nasal wash samples (1 mL each) were collected on days 2, 4, 6, 8, 10, and 12 post-infection. Viral loads were quantified by qPCR, and infectious virus titers in nasal washes were determined via titration assays.

Additionally, two weeks after the booster immunization, another set of three immunized minks and three PBS controls were intranasally challenged with the HRB25 strain (1×10^7^ PFU/1 mL per mink). On day 4 post-challenge, the minks were euthanized for necropsy. Euthanasia was performed prior to dissection using the following procedure: first, anesthesia was induced via intramuscular injection of Zoletil 50 (Virbac, France) at a dosage of 15 mg/kg. Once deep anesthesia was confirmed by the absence of reflexes and loss of consciousness, a potassium chloride solution was administered via intracardiac injection at a dosage of 150 mg/kg to induce cardiac arrest. Following the cessation of vital signs, organ samples (nasal turbinates, soft palate, tonsils, trachea, lungs, and small intestines) were collected. Following tissue homogenization, viral RNA was analyzed by qPCR, and infectious virus titers in tissue samples were simultaneously determined through titration. The remaining immunized minks in each group that were not subjected to viral challenge were euthanized. All euthanasia procedures complied with the *AVMA Guidelines for the Euthanasia of Animals*: *2020 Edition.*

### 2.11 Enzyme-linked immunospot assay

To evaluate T cell responses in rAd-S6P-immunized cats, an ELISPOT assay was performed using the Mabtech ELISPOT kit (catalog no. 3122-2H; Mabtech). Briefly, 14 days post-challenge, anticoagulated blood samples were collected from the cats (with three cats in each of the immunized and control groups), and peripheral blood mononuclear cells (PBMCs) were isolated using a feline lymphocyte separation buffer (TBD Science, Tianjin, China). Millipore 96-well HTS HA sterile plates (Millipore, USA) were coated with 15 μg/ml purified anti-feline IFN-γ antibody in 0.1 ml PBS and incubated at 4 °C for 8 hours.

Subsequently, 1×10^5^ PBMCs per well were seeded and co-cultured with live SARS-CoV-2 HRB25 strain at a multiplicity of infection (MOI) of 1 for 20 hours. After removing the cells, 0.1 ml of biotin-labeled anti-feline IFN-γ antibody was added and incubated for 1 hour, followed by the addition of 0.1 ml horseradish peroxidase (HRP)-conjugated streptavidin for another hour. Following the final wash steps, spots were developed using an AEC substrate kit (BD Pharmingen, San Diego, CA). Spot images were captured and counted using an AID ELISPOT reader (AID GmbH, Strassberg, Germany), and data were analyzed using Prism software (GraphPad, San Diego, CA). Each cat was tested in triplicate wells, and results are expressed as mean ± standard deviation (SD). Statistical analysis was performed using Student’s t-test.

### 2.12 Statistical analysis

The analyses were conducted using GraphPad Prism v.8.0.2. The outcomes of antibody responses in immunized animals, viral RNA copy numbers, and viral titers in nasal washes or animal organs were all evaluated via blinded assessment.

## 3 Results

### 3.1 Generation of recombinant adenovirus expressing SARS-CoV-2 *S* gene

**Figure 1:**
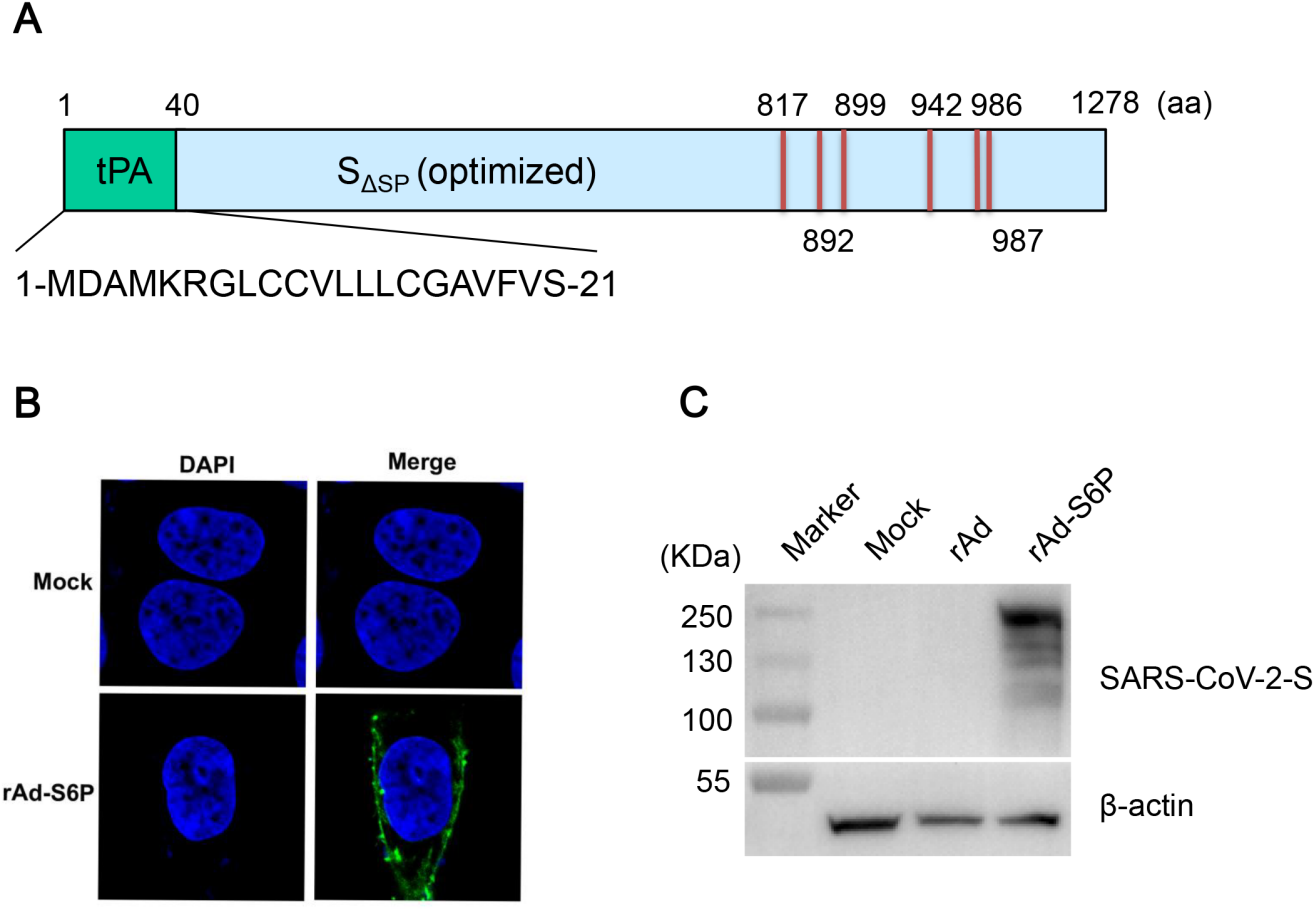
Construction of rAd-S6P and expression characterization of the SARS-CoV-2 spike protein. **(A)** Schematic representation of the *S* gene cassette in rAd-S6P: The gene was subjected to mammalian codon optimization, fused with a tissue plasminogen (tPA) signal peptide to replace the native signal sequence, and harbored six proline substitutions (F817P, A892P, A899P, A942P, K986P, V987P). **(B)** Indirect immunofluorescence assay (IFA) demonstrating S protein expression in AD-293 cells infected with rAd-S6P (green fluorescence). **(C)** Western blotting analysis confirming the expression of the S protein in rAd-S6P-infected AD-293 cells, using a specific anti-S polyclonal antibody.

The *S* gene (40–3819 nt) of SARS-CoV-2 strain HRB25 (GISAID accession EPI_ISL_467430) was artificially synthesized and subjected to mammalian codon optimization to enhance expression efficiency. Six specific amino acid residues (817F, 892A, 899A, 942A, 986K, and 987V) in the S protein were mutated to proline, and the native signal peptide (1–39 nt) of the *S* gene was replaced with the tissue plasminogen activator (tPA) signal peptide (1–66 nt) (**Figure 1A**). The modified *tPA-S6P* gene was cloned into a shuttle vector and integrated into the adenovirus genome via homologous recombination. The recombinant virus rAd-S6P was rescued by transfecting the recombined genome into AD-293 cells. PCR analysis confirmed the stable integration of the *S* gene in rAd-S6P (data not shown). Indirect immunofluorescence assay showed that the S protein was expressed in rAd-S6P-infected AD-293 cells, with predominant localization on the cell surface (**Figure 1B**). Western blot analysis validated the expression of the S protein in the recombinant adenovirus rAd-S6P (**Figure 1C**). The recombinant virus rAd-S6P was serially passaged to the third generation (P3). Viral genome extraction, PCR amplification, and Sanger sequencing confirmed the absence of mutations in the inserted *tPA-S6P* gene. The P3 virus was subsequently used as the immunogen for animal vaccination studies.

### 3.2 rAd-S6P elicits neutralizing antibodies and confers complete protection against SARS-CoV-2 challenge in mice

We first evaluated the immunogenicity of rAd-S6P in mice. Animals were monitored daily for 14 days post-immunization, with no abnormalities observed in general condition, food/water consumption, or activity.

**Figure 2:**
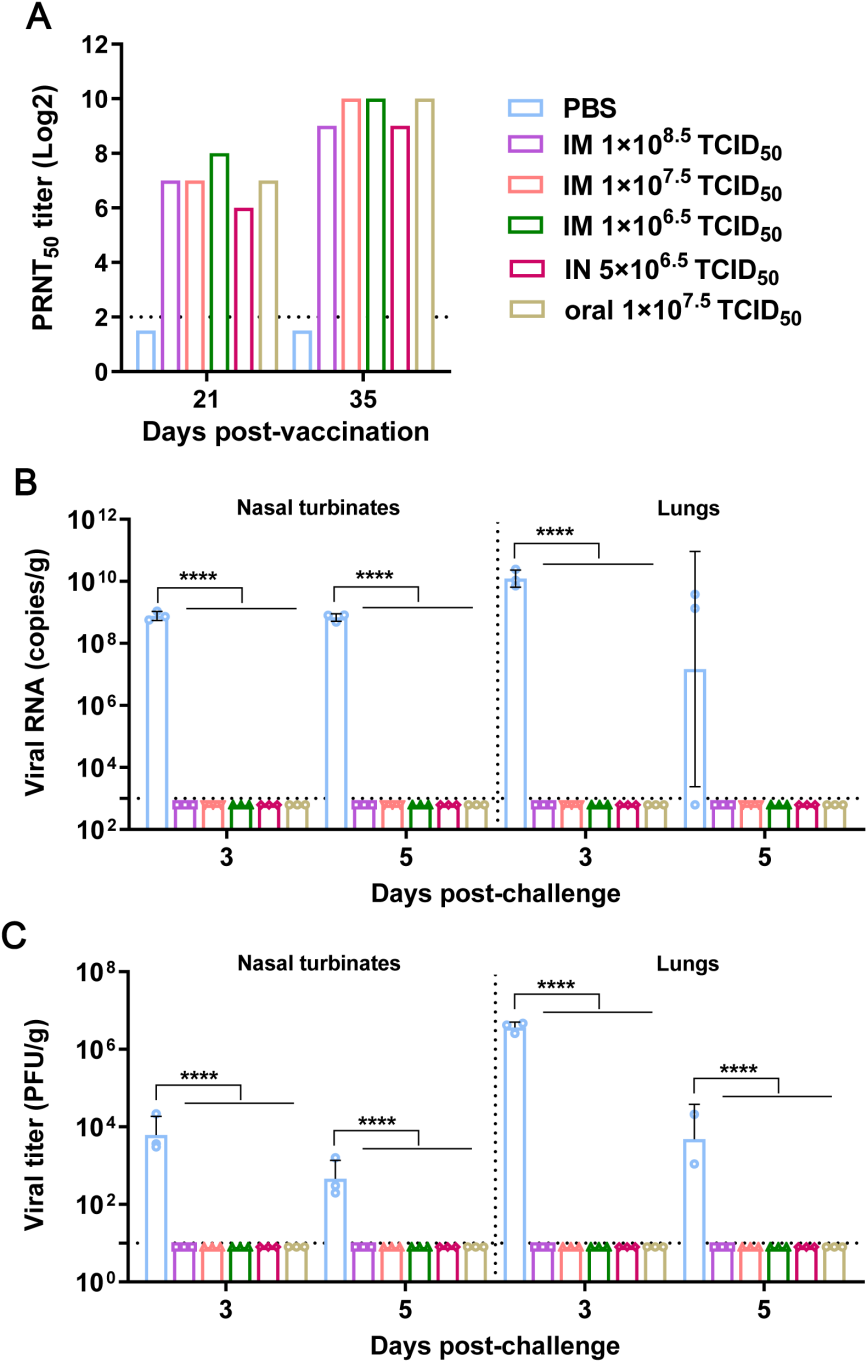
Humoral immune responses and protective efficacy of rAd-S6P in a mouse infection model. **(A)** SARS-CoV-2 neutralizing antibody titers in mouse sera collected at day 21 (1st dose) and day 35 (2nd dose), measured by PRNT. **(B)** Viral RNA copies in mouse nasal turbinates and lungs at days 3 and 5 post-challenge with 10^3.6^ PFU of mouse-adapted SARS-CoV-2 strain HRB26M. **(C)** Infectious virus titers in the same tissues, determined by plaque assay. Statistical significance was determined using two-way ANOVA. ****P < 0.0001..

Blood samples were collected from the infraorbital venous plexus at 3 weeks post-prime and 2 weeks post-boost. For each group, sera from five randomly selected mice were pooled. Serum was isolated and heat-inactivated at 56 °C for 30 minutes. Using the SARS-CoV-2 HRB25 strain, plaque reduction neutralization tests (PRNT) quantified neutralizing antibody titers. rAd-S6P-immunized mice developed neutralizing antibodies after the prime immunization, with titers further increasing following booster vaccination (**Figure 2A**). To evaluate dose effects on antibody induction, three IM dosage groups were tested; however, antibody levels did not increase with increasing dose.

We evaluated the protective efficacy of rAd-S6P in a pre-established mouse infection model, which is characterized by robust upper and lower respiratory tract replication of SARS-CoV-2 (Wang et al., 2020). Two weeks after booster immunization, six mice from each rAd-S6P-immunized group and six PBS-injected control mice were randomly selected for a virus challenge assay. Mice were challenged via nasal drip with the mouse-adapted SARS-CoV-2 strain HRB26M at a dose of 10^3.6^ PFU/100 μL/mouse. As shown in **Figures 2B, C**, high levels of viral RNA and infectious virus titers were detected in the nasal turbinates (NT) and lungs (LU) of PBS-inoculated mice on day 3 post-challenge. By contrast, no viral RNA or infectious virus was detected in the nasal turbinates or lungs of any rAd-S6P-immunized mice. Similarly, on day 5 post-infection, PBS-inoculated mice exhibited high viral RNA levels and infectious virus titers in these tissues, whereas all rAd-S6P-immunized mice remained negative for both markers. These results demonstrate that the immune response induced by rAd-S6P effectively protects mice against SARS-CoV-2 infection.

### 3.3 rAd-S6P elicits potent neutralizing antibodies and suppresses viral shedding in cats

**Figure 3:**
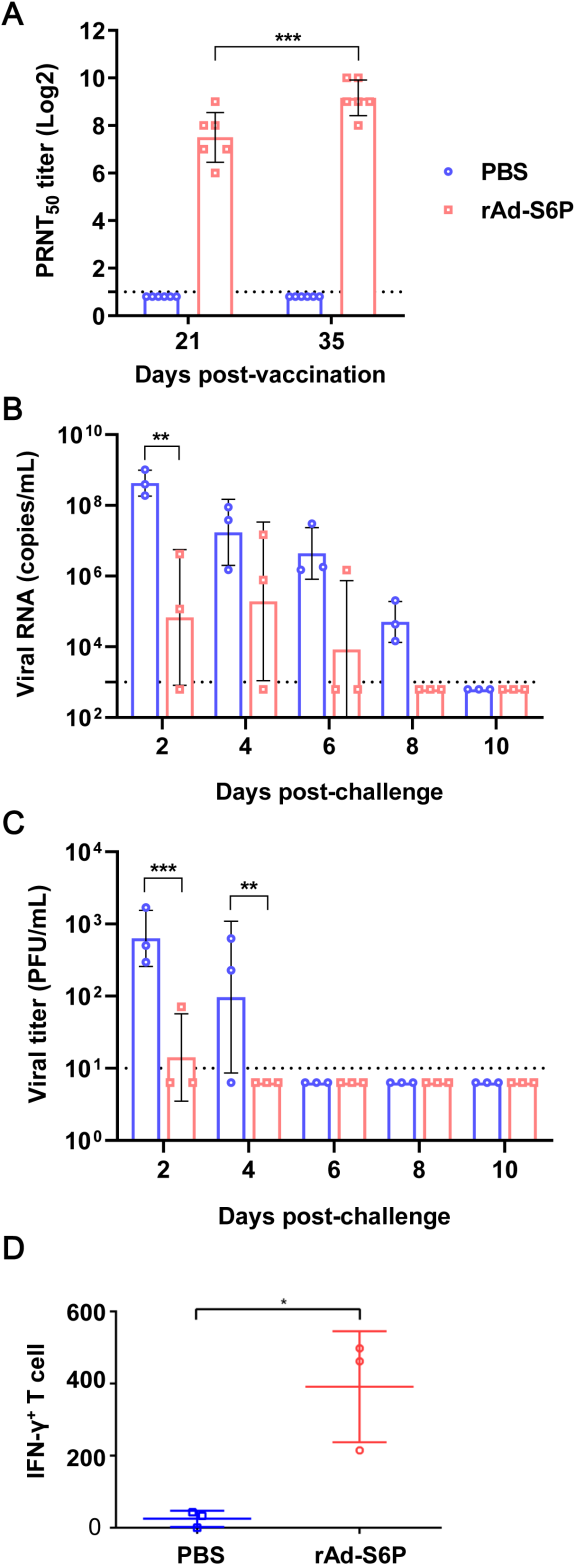
rAd-S6P elicits neutralizing antibodies and memory T cell responses against SARS-CoV-2, reducing upper respiratory tract viral replication in immunized cats. **(A)** Neutralizing antibody titers in cat sera measured by PRNT at day 21 (1st dose) and day 35 (2nd dose). **(B, C)** Viral RNA copies **(B)** and infectious virus titers **(C)** in nasal washes collected daily after intranasal challenge with 5×10^6^ PFU of SARS-CoV-2 strain HRB25. **(D)** IFN-γ-secreting T cell responses in cat PBMCs stimulated with HRB25, detected by IFN-γ ELISPOT assay 14 days post-challenge. Statistical significance was determined using two-way ANOVA. *P < 0.05, **P < 0.01, ***P < 0.001..

We evaluated the immunogenicity and protective efficacy of rAd-S6P in cats. After immunization, the cats were monitored daily for their general condition over a 14-day period. The assessment parameters included general condition, food and water intake, and activity levels, with no abnormalities detected throughout the observation period. Neutralizing antibodies were detected in all vaccinated animals following the first immunization, and titers were further enhanced after the second immunization (**Figure 3A**).

Two weeks after booster immunization, rAd-S6P-immunized cats and PBS-inoculated control cats were challenged via the IN lroute with the SARS-CoV-2 HRB25 strain at a dose of 5×10^6^ PFU/1 lmL per cat. Starting on day 2 post-challenge, nasal washes were collected every other day. As shown in **Figure 3B**, qPCR results revealed that high levels of viral RNA were detected in the nasal washes of all three control cats from day 2 to day 8 post-challenge. By contrast, viral RNA was detected in the nasal washes of two rAd-S6P-immunized cats from day 2 to day 4, and only one cat showed detectable viral RNA by day 6, with all viral RNA loads in immunized cats significantly lower than those in controls. On day 2 post-challenge, high titers of infectious virus were identified in the nasal washes of all three control cats, whereas only one rAd-S6P-immunized cat exhibited detectable infectious virus at a titer lower than that in the PBS-inoculated group. By day 4 post-challenge, high viral titers were found in the nasal washes of two control cats, while no infectious virus was detected in the nasal washes of any rAd-S6P-immunized cat (**Figure 3C**). These results demonstrate that the immune response induced by rAd-S6P effectively clears SARS-CoV-2 from cats and reduces viral shedding.

### 3.4 rAd-S6P elicits SARS-CoV-2-specific IFN-γ responses in cats

T cells play critical roles in clearing virus-infected cells through direct cytotoxicity and cytokine secretion. Moreover, the rapid expansion of vaccine-induced memory T cells is essential for boosting immunity and limiting COVID-19 pathogenesis and transmission (DiPiazza et al., 2021). To assess T cell responses, an ELISPOT assay was used to evaluate rAd-S6P-immunized cats 14 days post-viral challenge. As shown in **Figure 3D**, peripheral blood mononuclear cells (PBMCs) from rAd-S6P-immunized cats produced significantly higher levels of IFN-γ in response to HRB25 stimulation, whereas PBMCs from sham-immunized cats only exhibited baseline IFN-γ secretion. These results demonstrate that rAd-S6P elicits robust SARS-CoV-2-specific memory T cell responses in cats.

### 3.5 rAd-S6P elicits potent neutralizing antibodies and suppresses viral shedding in minks

**Figure 4:**
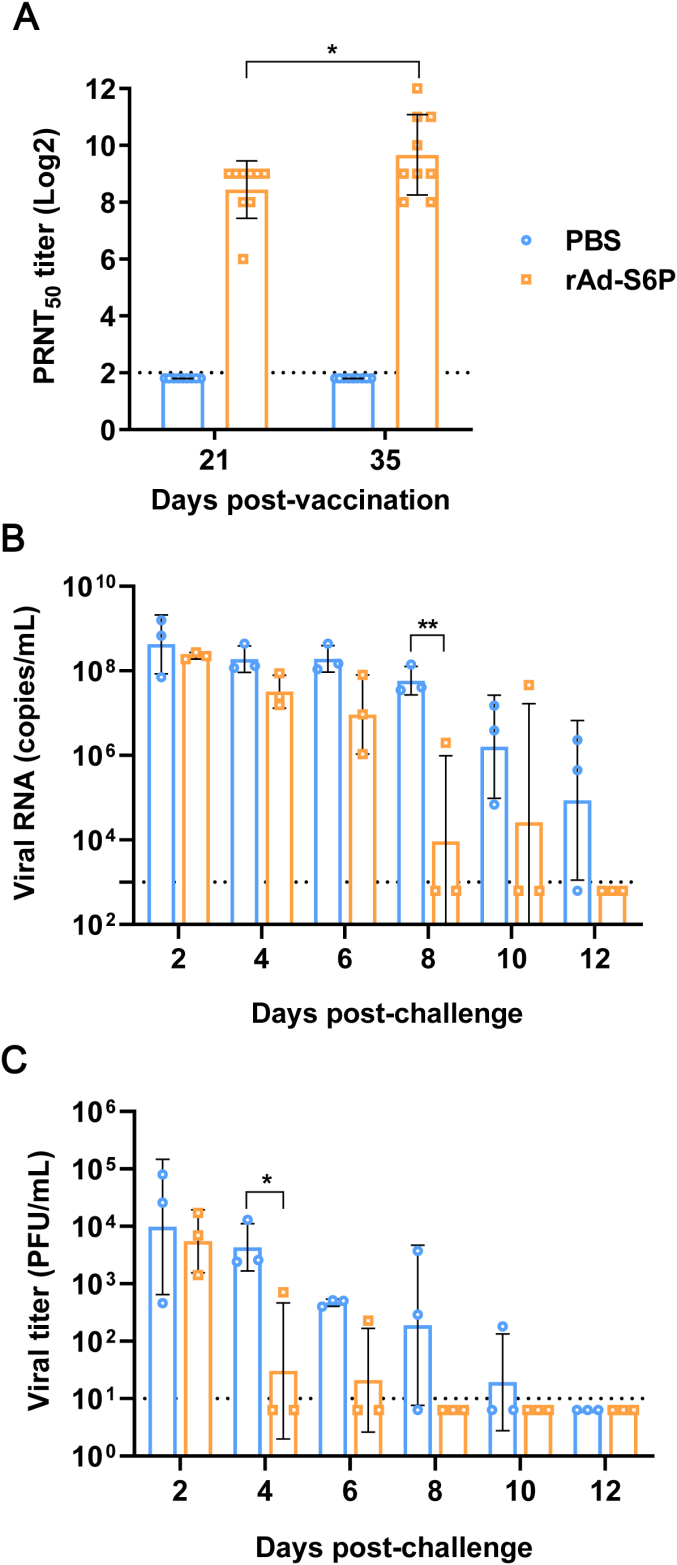
rAd-S6P elicits neutralizing antibodies and suppresses upper respiratory tract viral replication in immunized minks. **(A)** Neutralizing antibody titers in mink sera measured by PRNT at day 21 (1st dose) and day 35 (2nd dose). **(B, C)** Viral RNA copies **(B)** and infectious virus titers **(C)** in nasal washes collected daily after intranasal challenge with 1×10^7^ PFU of SARS-CoV-2 strain HRB25. Statistical significance was determined using two-way ANOVA. *P < 0.05, **P < 0.01..

To assess the immunogenicity of recombinant adenovirus rAd-S6P in minks, nine 13-month-old minks were immunized twice via IM injection with 1×10^9.5^ TCID_50_/1 mL per mink at 3-week intervals. Following immunization, the minks were subjected to daily monitoring over a 14-day period to evaluate their general condition, food and water intake, and activity levels. No abnormalities were detected throughout the observation period. Venous blood samples were collected 3 weeks post-initial immunization and 2 weeks post-booster immunization. Serum was isolated, inactivated in a 56°C water bath for 30 minutes, and subjected to a PRNT to quantify SARS-CoV-2 neutralizing antibody titers. Results showed that SARS-CoV-2 neutralizing antibodies were detectable in the serum of rAd-S6P-immunized minks 3 weeks after the initial immunization. Antibody titers significantly increased 2 weeks after the second immunization, whereas no neutralizing antibodies were detected in the serum of control minks (**Figure 4A**). These findings indicate that rAd-S6P exhibits excellent immunogenicity in minks, capable of eliciting neutralizing antibodies following a single immunization.

Using a mink infection model (Shuai et al., 2021), we evaluated the protective efficacy of recombinant adenovirus rAd-S6P against challenge with the SARS-CoV-2 HRB25 strain. Three rAd-S6P-immunized minks and three sham-control minks were intranasally challenged with 1×10^7^ PFU/1 mL of the virus per animal two weeks after booster immunization. Nasal wash samples were collected on days 2, 4, 6, 8, 10, and 12 post-challenge for viral load quantification by qPCR and titration of infectious virus. Results showed that viral RNA was detected in nasal washes of 3/3, 3/3, 3/3, 1/3, and 1/3 rAd-S6P-immunized minks on days 2, 4, 6, 8, and 10 post-challenge, respectively. In contrast, all sham-control minks (3/3) tested positive for viral RNA on days 2, 4, 6, 8, and 10 post-challenge, with 2 minks still showing detectable viral RNA in nasal washes on day 12 (**Figure 4B**). For infectious virus, titers were detected in nasal washes of 3/3, 1/3, and 1/3 rAd-S6P-immunized minks on days 2, 4, and 6 post-challenge, respectively. All sham-control minks (3/3) had detectable infectious virus on days 2, 4, and 6 post-challenge, whereas 2/3 and 1/3 sham-control minks exhibited infectious virus in nasal washes on days 8 and 10 post-challenge, respectively (**Figure 4C**).

**Figure 5:**
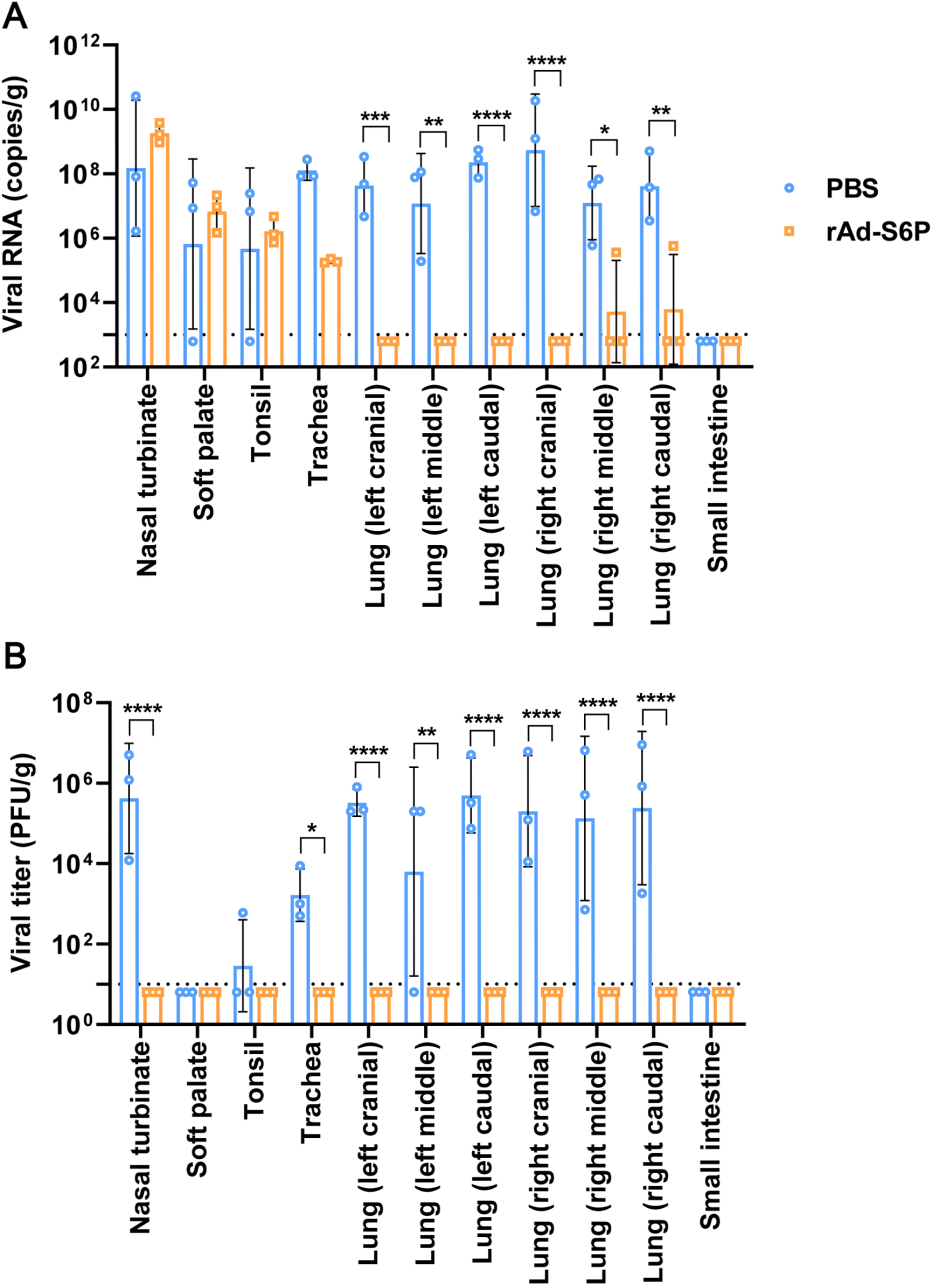
Viral replication profiles in organs of immunized minks post-challenge. **(A, B)** Viral RNA copies **(A)** and infectious virus titers **(B)** in nasal turbinate, soft palate, tonsil, trachea, lung, and small intestine collected from 3 minks per group euthanized at day 4 post-challenge with SARS-CoV-2 strain HRB25. Statistical significance was determined using two-way ANOVA.*P < 0.05, **P < 0.01, ***P < 0.001, ****P < 0.0001..

To evaluate the immunoprotective efficacy of the recombinant adenovirus rAd-S6P against SARS-CoV-2 in minks, three rAd-S6P-immunized animals and three sham-immunized control animals were intranasally challenged with 1×10^7^ PFU of the HRB25 viral strain (suspended in 1 mL) two weeks after the booster immunization. On day 4 post-infection, necropsies were performed on all three minks from each group. Tissues including nasal turbinates, soft palate, trachea, and lung lobes were excised, homogenized, and subjected to viral load quantification by qPCR. Concurrently, infectious virus titers in tissue homogenates were determined via standard plaque assays. The results demonstrated that, in comparison with the sham-control group, minks immunized with rAd-S6P exhibited a significant reduction in viral RNA load within the analyzed tissues, with the most pronounced decrease observed in lung tissues (**Figure 5A**). Notably, no infectious virus was recoverable from any organ samples collected from immunized animals (**Figure 5B**). These results collectively indicate that vaccination with rAd-S6P confers robust protective immunity in minks, effectively suppressing SARS-CoV-2 replication and preventing viral shedding.

## 4 Discussion

The development of veterinary vaccines against SARS-CoV-2 remains limited. Currently, there is no widely available commercial vaccine for animals against SARS-CoV-2. Subunit vaccines have been shown to induce robust immune responses (Morozov et al., 2023), while inactivated whole-virus vaccines can elicit neutralizing antibodies in dogs, cats, and other animal species (Chavda et al., 2021). Our previous research demonstrated that an adenovirus-vectored vaccine (rAd-S) protected ferrets against contact transmission (Wang et al., 2025).

Our previous comparative studies showed that replacing the native signal peptide of the S protein with that of tPA, combined with substituting the amino acid residues at positions 817, 892, 899, 942, 986, and 987 with proline, yields an optimized S6P protein. When used as an immunogen, this modified S6P protein elicits higher levels of neutralizing antibodies in mice compared to the unoptimized S protein.

Here we report the construction of an adenovirus-vectored SARS-CoV-2 vaccine (rAd-S6P) and evaluate its immunogenicity and protective efficacy in mouse, cat, and mink infection models. Our findings demonstrate that this vaccine candidate elicits robust neutralizing antibodies and memory T cell responses against SARS-CoV-2, thereby conferring complete protection to vaccinated animals. Based on the mouse immunization data, we found that: the antibody levels induced in mice immunized via intranasal (IN) or oral routes were comparable to those induced by intramuscular (IM) injection; furthermore, mice in the intranasal or oral immunization groups achieved complete protection following viral challenge, with no detectable virus in the nasal turbinates or lung tissues. This result suggests that future studies could further explore the immunogenicity and protective efficacy of intranasal or oral immunization in other animal models, such as cats and minks. Notably, this immunization strategy holds substantial application value for wildlife: by delivering immunogens into the wild as bait, wildlife can elicit SARS-CoV-2-specific antibodies via oral immunization. This not only reduces the risk of cross-species transmission of SARS-CoV-2 between animals and humans but also mitigates the potential threat of the virus undergoing adaptive mutations after infecting animal hosts and subsequently retransmitting to humans.

Cats, known to be highly susceptible to SARS-CoV-2 infection and efficient viral transmitters, showed significant suppression of infectious viral shedding from the upper respiratory tract following rAd-S6P immunization—findings indicating the vaccine’s potential to block viral transmission to cats and other susceptible hosts. Although cats immunized with rAd5-S6P achieved faster clearance of SARS-CoV-2 in nasal washes following viral challenge than PBS-immunized cats, they still could not prevent viral shedding from the upper respiratory tract. This observation may be associated with the immunization route. In the present study, while intramuscular immunization successfully induced neutralizing antibodies in cats, the antibody response at mucosal sites was presumably suboptimal. As a result, the virus could not be inhibited from establishing infection and replicating at the mucosal surfaces (Wang et al., 2025). In the future, we will continue to investigate whether mucosal immunization can prevent SARS-CoV-2 shedding from the upper respiratory tract in cats. Notably, despite minks’ high susceptibility to SARS-CoV-2 and efficient respiratory virus spread, rAd-S6P immunization induced potent neutralizing antibody responses. Following viral challenge, infectious virus was rapidly cleared from nasal washes, and no viable virus was detected in necropsied organ tissues. Given the established safety profile of adenoviral vectors and our preclinical data demonstrating both immunoprotection and transmission blockade, rAd-S6P represents a promising veterinary vaccine candidate for SARS-CoV-2 control.

Going forward, a multivalent broad-spectrum S protein antigen based on an adenoviral (Ad) vector could represent a viable strategy.

## Data Availability

The raw data supporting the conclusions of this article will be made available by the authors, without undue reservation.

## References

[B1] Chavda V. P. Feehan J. Apostolopoulos V. (2021). A veterinary vaccine for SARS-CoV-2: the first COVID-19 vaccine for animals. Vaccines 9, 631. doi: 10.3390/vaccines9060631, PMID: 34200587 PMC8228738

[B2] Dall Schmidt T. Mitze T. (2022). SARS-CoV-2 outbreaks on Danish mink farms and mitigating public health interventions. Eur. J. Public Health 32, 151–157. doi: 10.1093/eurpub/ckab182, PMID: 34623404 PMC8549281

[B3] Daniels J. B. Ratner J. J. Brown S. R. (1961). Plaque reduction, a sensitive test for eastern encephalitis antibody. Science. 133, 640–641. doi: 10.1126/science.133.3453.640, PMID: 13719624

[B4] Decaro N. Vaccari G. Lorusso A. Lorusso E. De Sabato L. Patterson E. I. . (2021). Possible human-to-dog transmission of SARS-CoV-2, Italy, 2020. Emerg. Infect. Dis. 27, 1981–1984. doi: 10.3201/eid2707.204959, PMID: 33979566 PMC8237870

[B5] DiPiazza A. T. Graham B. S. Ruckwardt T. J. (2021). T cell immunity to SARS-CoV-2 following natural infection and vaccination. Biochem. Biophys. Res. Commun. 538, 211–217. doi: 10.1016/j.bbrc.2020.10.060, PMID: 33190827 PMC7584424

[B6] Griffin B. D. Chan M. Tailor N. Mendoza E. J. Leung A. Warner B. M. . (2021). SARS-CoV-2 infection and transmission in the North American deer mouse. Nat. Commun. 12, 3612. doi: 10.1038/s41467-021-23848-9, PMID: 34127676 PMC8203675

[B7] Hale V. L. Dennis P. M. McBride D. S. Nolting J. M. Madden C. Huey D. . (2022). SARS-CoV-2 infection in free-ranging white-tailed deer. Nature. 602, 481–486. doi: 10.1038/s41586-021-04353-x, PMID: 34942632 PMC8857059

[B8] Hamer S. A. Pauvolid-Corrêa A. Zecca I. B. Davila E. Auckland L. D. Roundy C. M. . (2021). SARS-coV-2 infections and viral isolations among serially tested cats and dogs in households with infected owners in Texas, USA. Viruses 13. doi: 10.3390/v13050938, PMID: 34069453 PMC8159091

[B9] Hsieh C. L. Goldsmith J. A. Schaub J. M. DiVenere A. M. Kuo H. C. Javanmardi K. . (2020). Structure-based design of prefusion-stabilized SARS-CoV-2 spikes. Science. 369, 1501–1505. doi: 10.1126/science.abd0826, PMID: 32703906 PMC7402631

[B10] McAloose D. Laverack M. Wang L. Killian M. L. Caserta L. C. Yuan F. . (2020). From people to panthera: natural SARS-CoV-2 infection in tigers and lions at the Bronx Zoo. mBio 11. doi: 10.1128/mBio.02220-20, PMID: 33051368 PMC7554670

[B11] Morozov I. Gaudreault N. N. Trujillo J. D. Indran S. V. Cool K. Kwon T. . (2023). Preliminary study on the efficacy of a recombinant, subunit SARS-CoV-2 animal vaccine against virulent SARS-CoV-2 challenge in cats. Vaccines 11. doi: 10.3390/vaccines11121831, PMID: 38140233 PMC10747320

[B12] Oreshkova N. Molenaar R. J. Vreman S. Harders F. Oude Munnink B. B. Hakze-van der Honing R. W. . (2020). SARS-CoV-2 infection in farmed minks, the Netherlands, April and May 2020. Euro Surveill 25. doi: 10.2807/1560-7917.ES.2020.25.23.2001005, PMID: 32553059 PMC7403642

[B13] Oude Munnink B. B. Sikkema R. S. Nieuwenhuijse D. F. Molenaar R. J. Munger E. Molenkamp R. . (2021). Transmission of SARS-CoV-2 on mink farms between humans and mink and back to humans. Science. 371, 172–177. doi: 10.1126/science.abe5901, PMID: 33172935 PMC7857398

[B14] Pagani G. Lai A. Bergna A. Rizzo A. Stranieri A. Giordano A. . (2021). Human-to-cat SARS-CoV-2 transmission: case report and full-genome sequencing from an infected pet and its owner in Northern Italy. Pathogens 10. doi: 10.3390/pathogens10020252, PMID: 33672421 PMC7926546

[B15] Palmer M. V. Martins M. Falkenberg S. Buckley A. Caserta L. C. Mitchell P. K. . (2021). Susceptibility of white-tailed deer (Odocoileus virginianus) to SARS-CoV-2. J. Virol. 95. doi: 10.1128/JVI.00083-21, PMID: 33692203 PMC8139686

[B16] Reed L. J. Muench H. (1938). A simple method of estimating fifty per cent endpoints12. Am. J. Epidemiol. 27, 493–497. doi: 10.1093/oxfordjournals.aje.a118408

[B17] Shi J. Wen Z. Zhong G. Yang H. Wang C. Huang B. . (2020). Susceptibility of ferrets, cats, dogs, and other domesticated animals to SARS-coronavirus 2. Science. 368, 1016–1020. doi: 10.1126/science.abb7015, PMID: 32269068 PMC7164390

[B18] Shuai L. Zhong G. Yuan Q. Wen Z. Wang C. He X. . (2021). Replication, pathogenicity, and transmission of SARS-CoV-2 in minks. Natl. Sci. Rev. 8, nwaa291. doi: 10.1093/nsr/nwaa291, PMID: 34676095 PMC7798852

[B19] Sila T. Sunghan J. Laochareonsuk W. Surasombatpattana S. Kongkamol C. Ingviya T. . (2022). Suspected cat-to-human transmission of SARS-CoV-2, Thailand, July-September 2021. Emerging Infect. diseases. 28, 1485–1488. doi: 10.3201/eid2807.212605, PMID: 35666777 PMC9239874

[B20] Wang C. Shuai L. Zhong G. Wen Z. Liu R. Liu Q. . (2025). Transmission of SARS-CoV-2 between ferrets in presence of pre-existing immunity. J. Virol. 99, e0156625. doi: 10.1128/jvi.01566-25, PMID: 41186412 PMC12645953

[B21] Wang J. Shuai L. Wang C. Liu R. He X. Zhang X. . (2020). Mouse-adapted SARS-CoV-2 replicates efficiently in the upper and lower respiratory tract of BALB/c and C57BL/6J mice. Protein Cell. 11, 776–782. doi: 10.1007/s13238-020-00767-x, PMID: 32749592 PMC7401472

[B22] Wu S. Zhong G. Zhang J. Shuai L. Zhang Z. Wen Z. . (2020). A single dose of an adenovirus-vectored vaccine provides protection against SARS-CoV-2 challenge. Nat. Commun. 11, 4081. doi: 10.1038/s41467-020-17972-1, PMID: 32796842 PMC7427994

[B23] Yen H. L. Sit T. H. C. Brackman C. J. Chuk S. S. Y. Gu H. Tam K. W. S. . (2022). Transmission of SARS-CoV-2 delta variant (AY.127) from pet hamsters to humans, leading to onward human-to-human transmission: a case study. Lancet 399, 1070–1078. doi: 10.1016/S0140-6736(22)00326-9, PMID: 35279259 PMC8912929

[B24] Younes S. Younes N. Shurrab F. Nasrallah G. K . (2020). Severe acute respiratory syndrome coronavirus-2 natural animal reservoirs and experimental models: systematic review. Rev. Med. Virol. 31, e2196. doi: 10.1002/rmv.2196, PMID: 33206434 PMC7744864

[B25] Zhu F. C. Guan X. H. Li Y. H. Huang J. Y. Jiang T. Hou L. H. . (2020a). Immunogenicity and safety of a recombinant adenovirus type-5-vectored COVID-19 vaccine in healthy adults aged 18 years or older: a randomised, double-blind, placebo-controlled, phase 2 trial. Lancet. 396, 479–488. doi: 10.1016/S0140-6736(20)31605-6, PMID: 32702299 PMC7836858

[B26] Zhu F. Jin P. Zhu T. Wang W. Ye H. Pan H. . (2022). Safety and immunogenicity of a recombinant adenovirus type-5-vectored coronavirus disease 2019 (COVID-19) vaccine with a homologous prime-boost regimen in healthy participants aged >/=6 years: A randomized, double-blind, placebo-controlled, phase 2b trial. Clin. Infect. Dis. 75, e783–e791. doi: 10.1093/cid/ciab845, PMID: 34551104 PMC8522421

[B27] Zhu F. C. Li Y. H. Guan X. H. Hou L. H. Wang W. J. Li J. X. . (2020b). Safety, tolerability, and immunogenicity of a recombinant adenovirus type-5 vectored COVID-19 vaccine: a dose-escalation, open-label, non-randomised, first-in-human trial. Lancet. 395, 1845–1854. doi: 10.1016/S0140-6736(20)31208-3, PMID: 32450106 PMC7255193

