# Supplementary material for: Evaluation of the protective efficacy of a recombinant adenovirus-vectored SARS-CoV-2 vaccine candidate for veterinary use
Source: Front Cell Infect Microbiol. 2025 Dec 17;15:1714427. doi: 10.3389/fcimb.2025.1714427 (PMC12753914; doi:10.3389/fcimb.2025.1714427)
Supplement: Supplementary file 1 [file Table1.docx]

**Table 1. Neutralizing antibody titers in cat sera measured by plaque reduction neutralization test (PRNT).** Six cats were intramuscularly administered 1 mL of phosphate-buffered saline (PBS) to serve as the sham control group, while the remaining six cats received two intramuscular (IM) immunizations with rAd-S6P (1×10^9.5^ TCID_50_ per dose) at a 3-week interval. Neutralizing antibody titers in cat sera were measured by PRNT on day 21 (3 week after the first dose) and day 35 (2 weeks after the second dose).

| **Days post-vaccination** | PBS | | | | | |  | rAd-S6P | | | | | |
| --- | --- | --- | --- | --- | --- | --- | --- | --- | --- | --- | --- | --- | --- |
|  | 1 | 2 | 3 | 4 | 5 | 6 |  | 1 | 2 | 3 | 4 | 5 | 6 |
| 21 | ﹤2 | ﹤2 | ﹤2 | ﹤2 | ﹤2 | ﹤2 |  | 2^6^ | 2^7^ | 2^7^ | 2^9^ | 2^8^ | 2^8^ |
| 35 | ﹤2 | ﹤2 | ﹤2 | ﹤2 | ﹤2 | ﹤2 |  | 2^8^ | 2^9^ | 2^9^ | 2^10^ | 2^10^ | 2^9^ |

**Table 2. Viral RNA levels in nasal washes of cats.** Four weeks after the second immunization, both the immunized group and the control group (three cats per group) were intranasally (IN) challenged with SARS-CoV-2 strain HRB25 at a dose of 5×10^6^ PFU/mL. Nasal wash samples were collected on days 2, 4, 6, 8, and 10 post-challenge for viral RNA quantification.

| **Viral RNA levels in nasal washes of cats inoculated with SARS-CoV-2 isolate HRB25 (copies/mL, log_10_)** | | | | | | | |
| --- | --- | --- | --- | --- | --- | --- | --- |
| **Days post-challenge** | **PBS** | | |  | **rAd-S6P** | | |
|  | **1** | **2** | **3** |  | **1** | **2** | **3** |
| 2 | 8.60 | 8.28 | 9.01 |  | ﹤3 | 6.62 | 5.07 |
| 4 | 6.18 | 7.95 | 7.58 |  | ﹤3 | 5.89 | 7.17 |
| 6 | 6.26 | 6.18 | 7.48 |  | ﹤3 | ﹤3 | 6.17 |
| 8 | 4.64 | 4.16 | 5.31 |  | ﹤3 | ﹤3 | ﹤3 |
| 10 | ﹤3 | ﹤3 | ﹤3 |  | ﹤3 | ﹤3 | ﹤3 |

**Table 3. Viral titers in nasal washes of cats.** Four weeks after the second immunization, both the immunized group and the control group (three cats per group) were IN challenged with SARS-CoV-2 strain HRB25 at a dose of 5×10^6^ PFU/mL. Nasal wash samples were collected on days 2, 4, 6, 8, and 10 post-challenge for infectious virus quantification to monitor the dynamics of viral shedding.

| **Viral titers in nasal washes of cats inoculated with SARS-CoV-2 isolate HRB25 (PFU/mL, log_10_)** | | | | | | | |
| --- | --- | --- | --- | --- | --- | --- | --- |
| **Days post-challenge** | **PBS** | | |  | **rAd-S6P** | | |
|  | **1** | **2** | **3** |  | **1** | **2** | **3** |
| 2 | 2.47 | 2.70 | 3.23 |  | ﹤1 | 1.85 | ﹤1 |
| 4 | ﹤1 | 2.80 | 2.36 |  | ﹤1 | ﹤1 | ﹤1 |
| 6 | ﹤1 | ﹤1 | ﹤1 |  | ﹤1 | ﹤1 | ﹤1 |
| 8 | ﹤1 | ﹤1 | ﹤1 |  | ﹤1 | ﹤1 | ﹤1 |
| 10 | ﹤1 | ﹤1 | ﹤1 |  | ﹤1 | ﹤1 | ﹤1 |

**Table 4. Neutralizing antibody titers against SARS-CoV-2 in mink sera measured by PRNT.** Nine 13-month-old minks were intramuscularly inoculated with rAd-S6P at a dose of 1×10^9.5^ TCID_50_ per animal in a total volume of 1 mL. Additionally, nine minks in the control group received 1 mL of PBS via IM injection. A booster immunization was administered 3 weeks after the primary vaccination. Venous blood samples were collected at 3 weeks post-primary immunization and 2 weeks post-booster immunization. Neutralizing antibodies against SARS-CoV-2 were quantified using PRNT.

| **Days post-vaccination** | PBS | | | | | | | | |
| --- | --- | --- | --- | --- | --- | --- | --- | --- | --- |
|  | 1 | 2 | 3 | 4 | 5 | 6 | 7 | 8 | 9 |
| 21 | ﹤2 | ﹤2 | ﹤2 | ﹤2 | ﹤2 | ﹤2 | ﹤2 | ﹤2 | ﹤2 |
| 35 | ﹤2 | ﹤2 | ﹤2 | ﹤2 | ﹤2 | ﹤2 | ﹤2 | ﹤2 | ﹤2 |

| **Days post-vaccination** | rAd-S6P | | | | | | | | |
| --- | --- | --- | --- | --- | --- | --- | --- | --- | --- |
|  | 1 | 2 | 3 | 4 | 5 | 6 | 7 | 8 | 9 |
| 21 | 2^6^ | 2^8^ | 2^9^ | 2^9^ | 2^9^ | 2^8^ | 2^9^ | 2^9^ | 2^9^ |
| 35 | 2^8^ | 2^9^ | 2^9^ | 2^11^ | 2^8^ | 2^11^ | 2^9^ | 2^10^ | 2^12^ |

**Table 5. Viral RNA levels in nasal washes of minks.** Three rAd-S6P-immunized minks and three PBS-inoculated control minks were IN challenged with the SARS-CoV-2 strain HRB25 two weeks after booster immunization, at a dose of 1×10^7^ PFU per animal in a total volume of 1 mL. Nasal wash samples (1 mL each) were collected on days 2, 4, 6, 8, 10, and 12 post-challenge. Viral RNA was quantified by quantitative polymerase chain reaction (qPCR).

| **Viral RNA levels in nasal washes of minks inoculated with SARS-CoV-2 isolate HRB25 (copies/mL, log_10_)** | | | | | | | |
| --- | --- | --- | --- | --- | --- | --- | --- |
| **Days post-challenge** | **PBS** | | |  | **rAd-S6P** | | |
|  | **1** | **2** | **3** |  | **1** | **2** | **3** |
| 2 | 9.20 | 8.83 | 7.85 |  | 8.44 | 8.28 | 8.35 |
| 4 | 8.64 | 8.12 | 8.07 |  | 7.94 | 7.39 | 7.19 |
| 6 | 8.17 | 8.64 | 8.04 |  | 6.97 | 7.90 | 6.03 |
| 8 | 7.61 | 8.15 | 7.54 |  | ﹤3 | 6.30 | ﹤3 |
| 10 | 6.59 | 7.18 | 4.84 |  | ﹤3 | 7.66 | ﹤3 |
| 12 | 5.65 | 6.36 | ﹤3 |  | ﹤3 | ﹤3 | ﹤3 |

**Table 6. Viral titers in nasal washes of minks.** Three rAd-S6P-immunized minks and three PBS-inoculated control minks were IN challenged with the SARS-CoV-2 strain HRB25 two weeks after booster immunization, at a dose of 1×10^7^ PFU per animal in a total volume of 1 mL. Nasal wash samples (1 mL each) were collected on days 2, 4, 6, 8, 10, and 12 post-challenge. Infectious virus titers in nasal washes were determined via plaque-forming unit (PFU) assay.

| **Viral titers in nasal washes of minks inoculated with SARS-CoV-2 isolate HRB25 (PFU/mL, log_10_)** | | | | | | | |
| --- | --- | --- | --- | --- | --- | --- | --- |
| **Days post-challenge** | **PBS** | | |  | **rAd-S6P** | | |
|  | **1** | **2** | **3** |  | **1** | **2** | **3** |
| 2 | 4.90 | 4.41 | 2.66 |  | 3.84 | 4.23 | 3.15 |
| 4 | 4.11 | 3.38 | 3.41 |  | 2.85 | ﹤1 | ﹤1 |
| 6 | 2.60 | 2.71 | 2.70 |  | 2.36 | ﹤1 | ﹤1 |
| 8 | ﹤1 | 3.57 | 2.46 |  | ﹤1 | ﹤1 | ﹤1 |
| 10 | ﹤1 | 2.26 | ﹤1 |  | ﹤1 | ﹤1 | ﹤1 |
| 12 | ﹤1 | ﹤1 | ﹤1 |  | ﹤1 | ﹤1 | ﹤1 |

**Table 7. Viral RNA levels in organs of minks.** Two weeks after the booster immunization, another set of three rAd-S6P-immunized minks and three PBS-inoculated control minks were IN challenged with the SARS-CoV-2 strain HRB25 at a dose of 1×10^7^ PFU per animal in a total volume of 1 mL. On day 4 post-challenge, the minks were euthanized for necropsy. Following the cessation of vital signs, organ samples (nasal turbinates, soft palate, tonsils, trachea, lungs, and small intestines) were collected. After tissue homogenization, viral RNA was analyzed by qPCR.

| **Viral RNA levels in organs of minks inoculated with SARS-CoV-2 isolate HRB25 (copies/mL, log_10_)** | | | | | | | |
| --- | --- | --- | --- | --- | --- | --- | --- |
| **Organs** | **PBS** | | |  | **rAd-S6P** | | |
|  | **1** | **2** | **3** |  | **1** | **2** | **3** |
| Nasal turbinate | 10.41 | 7.91 | 6.22 |  | 9.23 | 8.97 | 9.58 |
| Soft palate | 7.72 | 6.94 | ﹤3 |  | 6.98 | 6.17 | 7.33 |
| Tonsil | 7.39 | 6.83 | ﹤3 |  | 6.12 | 5.86 | 6.67 |
| Trachea | 8.45 | 7.93 | 7.92 |  | 5.36 | 5.26 | 5.24 |
| Lung (left cranial) | 6.67 | 8.53 | 7.68 |  | ﹤3 | ﹤3 | ﹤3 |
| Lung (left middle) | 8.05 | 5.28 | 7.89 |  | ﹤3 | ﹤3 | ﹤3 |
| Lung (left caudal) | 8.74 | 7.87 | 8.46 |  | ﹤3 | ﹤3 | ﹤3 |
| Lung (right cranial) | 9.09 | 6.83 | 10.26 |  | ﹤3 | ﹤3 | ﹤3 |
| Lung (right middle) | 7.66 | 7.83 | 5.77 |  | ﹤3 | ﹤3 | 5.55 |
| Lung (right caudal) | 7.57 | 8.7 | 6.54 |  | ﹤3 | ﹤3 | 5.75 |
| Small intestine | ﹤3 | ﹤3 | ﹤3 |  | ﹤3 | ﹤3 | ﹤3 |

**Table 8. Viral titers in organs of minks.** Two weeks after the booster immunization, another set of three rAd-S6P-immunized minks and three PBS-inoculated control minks were IN challenged with the SARS-CoV-2 strain HRB25 at a dose of 1×10^7^ PFU per animal in a total volume of 1 mL. On day 4 post-challenge, the minks were euthanized for necropsy. Following the cessation of vital signs, organ samples (nasal turbinates, soft palate, tonsils, trachea, lungs, and small intestines) were collected. After tissue homogenization, infectious virus titers in tissue samples were determined via PFU.

| **Viral titers in organs of minks inoculated with SARS-CoV-2 isolate HRB25 (PFU/mL, log_10_)** | | | | | | | |
| --- | --- | --- | --- | --- | --- | --- | --- |
| **Organs** | **PBS** | | |  | **rAd-S6P** | | |
|  | **1** | **2** | **3** |  | **1** | **2** | **3** |
| Nasal turbinate | 6.08 | 4.08 | 6.70 |  | ﹤1 | ﹤1 | ﹤1 |
| Soft palate | ﹤1 | ﹤1 | ﹤1 |  | ﹤1 | ﹤1 | ﹤1 |
| Tonsil | 2.78 | ﹤1 | ﹤1 |  | ﹤1 | ﹤1 | ﹤1 |
| Trachea | 2.70 | 3.00 | 3.94 |  | ﹤1 | ﹤1 | ﹤1 |
| Lung (left cranial) | 5.34 | 5.90 | 5.30 |  | ﹤1 | ﹤1 | ﹤1 |
| Lung (left middle) | 5.30 | ﹤1 | 5.30 |  | ﹤1 | ﹤1 | ﹤1 |
| Lung (left caudal) | 6.70 | 4.87 | 5.51 |  | ﹤1 | ﹤1 | ﹤1 |
| Lung (right cranial) | 6.78 | 5.08 | 4.04 |  | ﹤1 | ﹤1 | ﹤1 |
| Lung (right middle) | 6.81 | 5.70 | 2.85 |  | ﹤1 | ﹤1 | ﹤1 |
| Lung (right caudal) | 3.26 | 6.95 | 5.92 |  | ﹤1 | ﹤1 | ﹤1 |
| Small intestine | ﹤1 | ﹤1 | ﹤1 |  | ﹤1 | ﹤1 | ﹤1 |
